# Hepatosplanchnic perfusion changes following isolated abdominal trauma and surgery: A scoping review of evidence dominated by pneumoperitoneum models

**DOI:** 10.14814/phy2.71051

**Published:** 2026-08-13

**Authors:** Vid Pivec, Andrej Bergauer, Anna Hawliczek, Nandu Goswami

**Affiliations:** ^1^ Clinical Department for Abdominal and General Surgery University Clinical Centre Maribor Maribor Slovenia; ^2^ Gravitational Physiology and Medicine Research Unit, Division of Physiology and Pathophysiology, Otto Loewi Research Center of Vascular Biology, Immunity and Inflammation Medical University of Graz Graz Austria; ^3^ Department for General and Visceral Surgery LKH Südweststeiermark Wagna Austria; ^4^ Center for Space and Aviation Health, College of Medicine Mohammed Bin Rashid University of Medicine and Health Sciences, Dubai Health Dubai UAE

**Keywords:** abdominal injuries/physiopathology [MeSH], hemodynamics [MeSH], intra‐abdominal pressure/physiology [MeSH], major abdominal surgery, mesenteric arteries/physiology [MeSH], microcirculation, portal vein/physiology [MeSH], scoping review, splanchnic circulation [MeSH], systematic review

## Abstract

The hepatosplanchnic vascular response to isolated abdominal organ injury remains poorly defined. The aim of this review was to synthesize the available evidence on macrovascular hepatosplanchnic flow and organ‐specific microvascular perfusion following isolated abdominal injury or surgery. PubMed/MEDLINE was searched through 11 May 2026 for human and animal studies reporting visceral hemodynamic changes after abdominal trauma, experimental injury, or surgery. Systematic‐review methods included structured study selection, data extraction, and design‐specific risk‐of‐bias assessment within a PRISMA‐based scoping‐review framework. Owing to heterogeneity, findings were synthesized narratively. Twenty‐six studies were included: 14 human and 12 animal studies. Nineteen investigated pneumoperitoneum or elevated intra‐abdominal pressure, four major abdominal surgery, and three other models. Perfusion reductions became more frequent with increasing pressure and were significantly more common at >15 than <10 mmHg (*p* = 0.031). Higher pressures also impaired compensatory mechanisms, including the hepatic arterial buffer response. After major surgery, portal and total hepatosplanchnic flow generally increased early postoperatively, while gastric mucosal perfusion deteriorated, indicating macro–microcirculatory dissociation. Only one study directly examined primary intra‐abdominal injury and assessed microcirculation alone. Evidence remains indirect, heterogeneous, and methodologically limited. Studies combining serial macrovascular and organ‐specific microvascular measurements are required to define the hepatosplanchnic response.

## 1 INTRODUCTION

The hepatovisceral circulation plays a central role in maintaining systemic blood pressure and organ oxygenation during stress. Extensive research has examined the effects of systemic stressors—such as exercise (Knight et al., 2017; Osada et al., 1999; Osada et al., 2009), polytrauma (Gottlieb et al., 1984; Malbrain et al., 2004), sepsis (Malbrain et al., 2004; Schmidt & Stallmach, 2007), and shock (Hinghofer‐Szalkay et al., 2008; Holm et al., 2006)—on visceral hemodynamics. However, the normal splanchnic vascular response to isolated abdominal organ and/or tissue injury (including surgical and experimental abdominal trauma) remains poorly characterized. In particular, it is unclear how blood flow through the superior mesenteric artery (SMA), celiac trunk (CT), and portal vein (PV) changes after injury or surgical resection of intra‐abdominal structures. Furthermore, it remains unclear how splanchnic vascular flow changes at the level of individual organs. This lack of knowledge limits our understanding of basic physiology and may have implications for perioperative management and critical care.

The objective of this scoping review was to identify and synthesize available evidence on changes in portal venous and arterial visceral blood flow following isolated abdominal organ and/or tissue injury, whether traumatic or iatrogenic (surgical). Specifically, we aimed to evaluate how blood flow in the SMA, CT, and PV at the macrovascular level is affected by such injuries or resections. In addition, we aimed to assess whether the splanchnic vascular response differs at the organ‐specific microvascular level.

## 2 METHODS

The review protocol was defined a priori and specified the study objectives, eligibility criteria, and methodological framework (Appendix S1).

### 2.1 Eligibility criteria

Included in the review were studies in humans and animals that reported visceral hemodynamic changes after isolated abdominal interventions, including trauma models or surgical procedures. Both experimental and clinical studies were eligible. Only articles in English, German and Polish were included. Exclusion criteria were non‐original studies (reviews, editorials), studies focusing on systemic trauma, and studies investigating liver resection or transplantation because of the liver's integral role in the portal venous system and hepatic arterial buffer response (HABR).

### 2.2 Information sources

A systematic search was conducted in PubMed/Medline. The most recent search update was performed on 11th of May 2026.

### 2.3 Search strategy

The search strategy combined Medical Subject Headings (MeSH) and keywords/phrases related to abdominal organ and tissue injury and to splanchnic or hepatic circulation.

- MeSH terms for abdominal interventions and injuries: “Wounds and Injuries,” “General Surgery,” “Colorectal Surgery,” “Hand‐Assisted Laparoscopy,” “Endoscopy, Digestive System,” “Gynecologic Surgical Procedures,” “Reoperation,” “Laparoscopy,” “Endoscopy,” “Gastrointestinal,” “Cytoreduction Surgical Procedures,” “Minimally Invasive Surgical Procedures,” “Gastroscopy,” “Esophagoscopy,” “Duodenoscopy,” “Colonoscopy.”
- Keywords for abdominal interventions and injuries: “surgery,” “general surgery,” “cholecystectomy,” “colon surgery,” “right hemicolectomy,” “left hemicolectomy,” “colorectal surgery,” “rectal surgery,” “total colectomy,” “subtotal colectomy,” “total coloproctectomy,” “segmental colon surgery,” “gastrectomy,” “subtotal gastrectomy,” “total gastrectomy,” “pancreatectomy,” “subtotal pancreatectomy,” “Whipple procedure”, “blunt trauma”, “blast trauma”, “gunshot injuries”, “penetrating trauma”, “abdominal stab wound.”
- MeSH terms and keywords for circulation: “Splanchnic Circulation,” “Splanchnic Circulation/physiology,” “Liver Circulation/physiology,” “Hyperemia,” and “reactive hyperemia.”

Search terms were combined in various Boolean combinations to maximize sensitivity.

### 2.4 Selection process

The search identified 1663 records. After title screening and exclusion of non‐original studies, 89 articles were retained for abstract review. When eligibility remained uncertain, the full text was assessed. This process yielded 42 full‐text articles, of which 26 studies were finally included. Study selection was performed independently by two reviewers, with disagreements resolved by discussion.

### 2.5 Data collection process

Data were extracted systematically from each included study into a structured evidence table. Extracted items included author, year of publication, study design, study model, experimental setting, measurement technique, type of intervention or injury model, sample size, study duration and measurement time points, macrocirculatory outcomes, microcirculatory outcomes, magnitude of effect, and the main physiological finding. Macrocirculatory outcomes were defined as changes in flow within named hepatosplanchnic vessels or global organ inflow/outflow, whereas microcirculatory outcomes were defined as changes at the tissue, mucosal, sinusoidal, or cellular perfusion level. Risk‐of‐bias assessment was recorded separately according to the appropriate tool for each study design, including the tool used, domain‐level judgments, and the overall risk‐of‐bias judgment.

### 2.6 Data items

The primary outcomes were changes in blood flow through the portal vein (PV), superior mesenteric artery (SMA), celiac trunk (CT), and total hepatic blood flow (THBF) following isolated abdominal injury, surgical intervention, experimental abdominal trauma, or pneumoperitoneum. Microcirculatory outcomes included organ‐specific tissue perfusion, hepatic or intestinal microvascular flow, gastric intramucosal pH (pHi), gastric mucosal PCO_2_ gap, tissue oxygenation, sinusoidal perfusion, leukocyte or platelet endothelial adhesion, Kupffer‐cell activation, and reperfusion‐related phenomena. Secondary data items included intra‐abdominal pressure (IAP), hepatic arterial buffer response (HABR), hepatic arterial, portal venous, and mesenteric flow parameters, organ‐specific microcirculatory alterations, gastric intramucosal pH (pHi), gastric mucosal PCO_2_ gap, tissue oxygenation, reperfusion phenomena, and device‐specific hemodynamic measurements.

### 2.7 Study risk of bias assessment

Risk of bias was assessed formally according to study design. Randomized clinical trials were evaluated using the Cochrane Risk of Bias 2 (RoB 2) (Sterne et al., 2019) tool, non‐randomized human studies using the Risk Of Bias In Non‐randomized Studies of Interventions (ROBINS‐I) (Sterne et al., 2016) tool, and experimental animal studies using SYRCLE's (Hooijmans et al., 2014) Risk of Bias tool. Domain‐level judgments and overall risk‐of‐bias judgments were recorded for each study. Because different tools were required for different study designs, risk‐of‐bias assessments were reported separately by assessment tool and were not combined into a single numerical quality score.

### 2.8 Effect measures

Due to heterogeneity in study design, species, interventions, measurement techniques, and reporting formats, no single effect measure could be applied across all included studies. Where baseline and intervention or postoperative values were available, macrovascular outcomes were summarized as relative percentage change from baseline, including portal venous blood flow, superior mesenteric arterial blood flow, hepatic arterial flow, and total hepatic or hepatovisceral blood flow. Pneumoperitoneum studies were additionally stratified by intra‐abdominal pressure level. Microvascular outcomes were summarized as direction and magnitude of change from baseline where possible, but were not pooled because of substantial heterogeneity in measurement techniques, including gastric tonometry, laser Doppler flowmetry, tissue oxygen tension, intravital microscopy, and laser speckle contrast imaging.

### 2.9 Synthesis methods

Data were synthesized narratively and, where possible, semi‐quantitatively. Studies were grouped by study model (human vs. animal), type of abdominal injury or intervention (pneumoperitoneum/IAP elevation, insufflation gas type, laparoscopic or open surgical procedure, portal triad clamping, aortic cross‐clamping, peritonitis, or major abdominal surgery), and level of perfusion assessment (macrovascular vs. microvascular).

For studies reporting comparable baseline and intervention or postoperative values, changes in portal venous blood flow, superior mesenteric arterial blood flow, hepatic arterial flow, total hepatic or hepatovisceral blood flow, and selected microvascular parameters were summarized as relative percentage change from baseline. Pneumoperitoneum studies were additionally stratified according to intra‐abdominal pressure level where possible.

Because of substantial heterogeneity in species, interventions, measurement devices, pressure levels, time points, and reported outcomes, no formal pooled meta‐analysis was performed. Instead, quantitative findings were presented descriptively as direction and magnitude of change, together with the main physiological interpretation.

### 2.10 Reporting bias assessment

Following study‐level risk‐of‐bias assessment, separate risk‐of‐bias traffic‐light plots were constructed according to study design and assessment tool. One RoB 2 traffic‐light plot was generated for the three randomized controlled studies, one ROBINS‐I traffic‐light plot for the 11 non‐randomized human studies, and one SYRCLE traffic‐light plot for the 12 experimental animal studies. These plots were used to visualize domain‐level and overall risk‐of‐bias judgments across the included studies.

### 2.11 Certainty assessment

No formal GRADE certainty‐of‐evidence assessment was undertaken because the review addressed heterogeneous physiological responses rather than a single therapeutic intervention or predefined pooled treatment effect. The certainty of the available evidence was considered limited because of methodological heterogeneity, indirectness of experimental and animal models, variability in measurement techniques and time points, small sample sizes, and the limited number of studies available for each outcome.

## 3 RESULTS

### 3.1 Study selection

**FIGURE 1:**
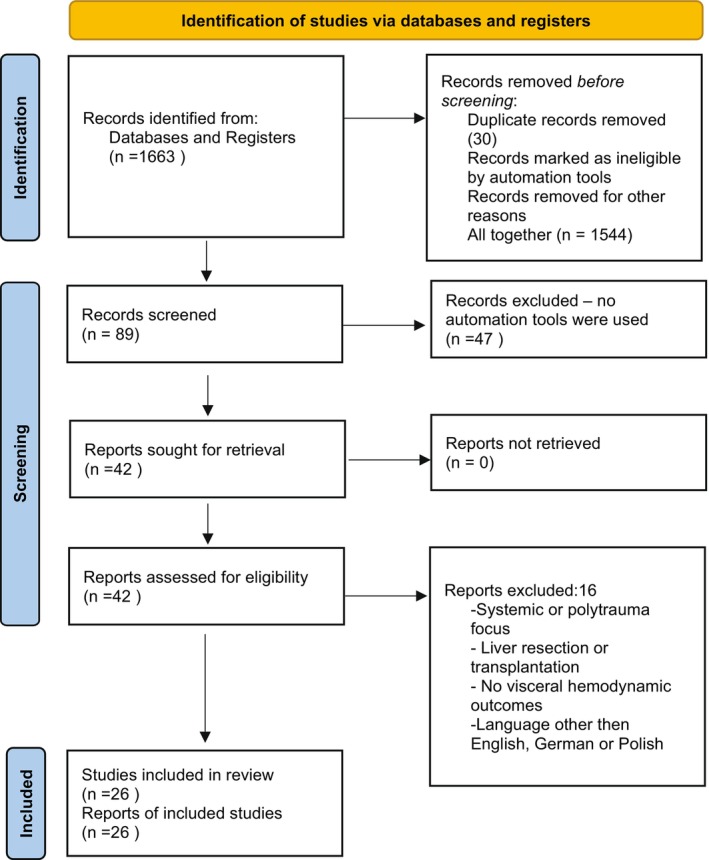
PRISMA flowchart.

The systematic search identified 1663 records. After title screening and exclusion of non‐original studies, 89 articles were considered potentially relevant. Following abstract review and full‐text assessment where required, 42 articles were examined in detail. Ultimately, 26 studies met the eligibility criteria and were included in this review. A flow diagram of the study selection process is presented in Figure 1 (PRISMA flowchart (Page et al., 2021; Tricco et al., 2018)).

### 3.2 Characteristics of sources of evidence

Of the 26 included studies, 14 were human studies and 12 were prospective experimental animal studies using swine/pig, rat, and dog models. Among the human studies, 11 were non‐randomized observational or comparative physiological studies and 3 were randomized controlled trials. The mean sample size was 34.1 ± 26.1 (SD) for the human cohort and 17.3 ± 9.2 (SD) for the animal studies.

The majority of human studies investigated the effect of elevated intra‐abdominal pressure (IAP) during laparoscopic procedures, while additional studies assessed major abdominal surgery, pancreatic surgery, high‐risk abdominal surgery, and aortic cross‐clamping. Animal studies predominantly applied controlled IAP elevation or pneumoperitoneum‐based models, with additional models including fluid‐induced IAP elevation, portal triad clamping, and polymicrobial peritonitis.

A variety of relevant hepatosplanchnic perfusion measurement principles were employed, resulting in 29 measurement‐method entries across the 26 included studies. These included implanted electromagnetic, ultrasonic, or transonic flow probes (Blobner et al., 1998; Diebel et al., 1992; Ishizaki et al., 1993a; Ishizaki et al., 1993b; Junghans et al., 1997; Richter et al., 2001) (*n* = 6), transabdominal, transesophageal and laparoscopic Doppler ultrasound techniques (Bella et al., 2004; Jakimowicz et al., 1998; Kaffarnik et al., 2018; Meierhenrich et al., 2005; Sato et al., 2000) (*n* = 5), gastric tonometry (Collange et al., 2013; Eleftheriadis et al., 1996; Knolmayer et al., 1998; Koivusalo et al., 1997; Poeze et al., 2002; Reyad et al., 2013; Thaler et al., 1996) (*n* = 7), laser Doppler or laser flowmetry techniques (Ali et al., 2005; Eleftheriadis et al., 1996; Nsadi et al., 2011; Schilling et al., 1997) (*n* = 4), ICG‐based techniques (Kaffarnik et al., 2018; Odeberg et al., 1998; Poeze et al., 2002; Suojaranta‐Ylinen et al., 2000) (*n* = 4), γ‐labeled microspheres (Schäfer et al., 2001) (*n* = 1), in vivo fluorescence microscopy (Nickkholgh et al., 2008) (*n* = 1), and laser speckle contrast imaging (*n* = 1) (Ding et al., 2014). Three studies combined more than one relevant measurement principle: Eleftheriadis et al. (1996) combined intraparenchymal laser Doppler measurement with gastric tonometry, Kaffarnik et al. (2018) combined Doppler ultrasound with ICG‐based assessment, and Poeze et al. (2002) combined continuous ICG infusion with gastric tonometry.

**FIGURE 2:**
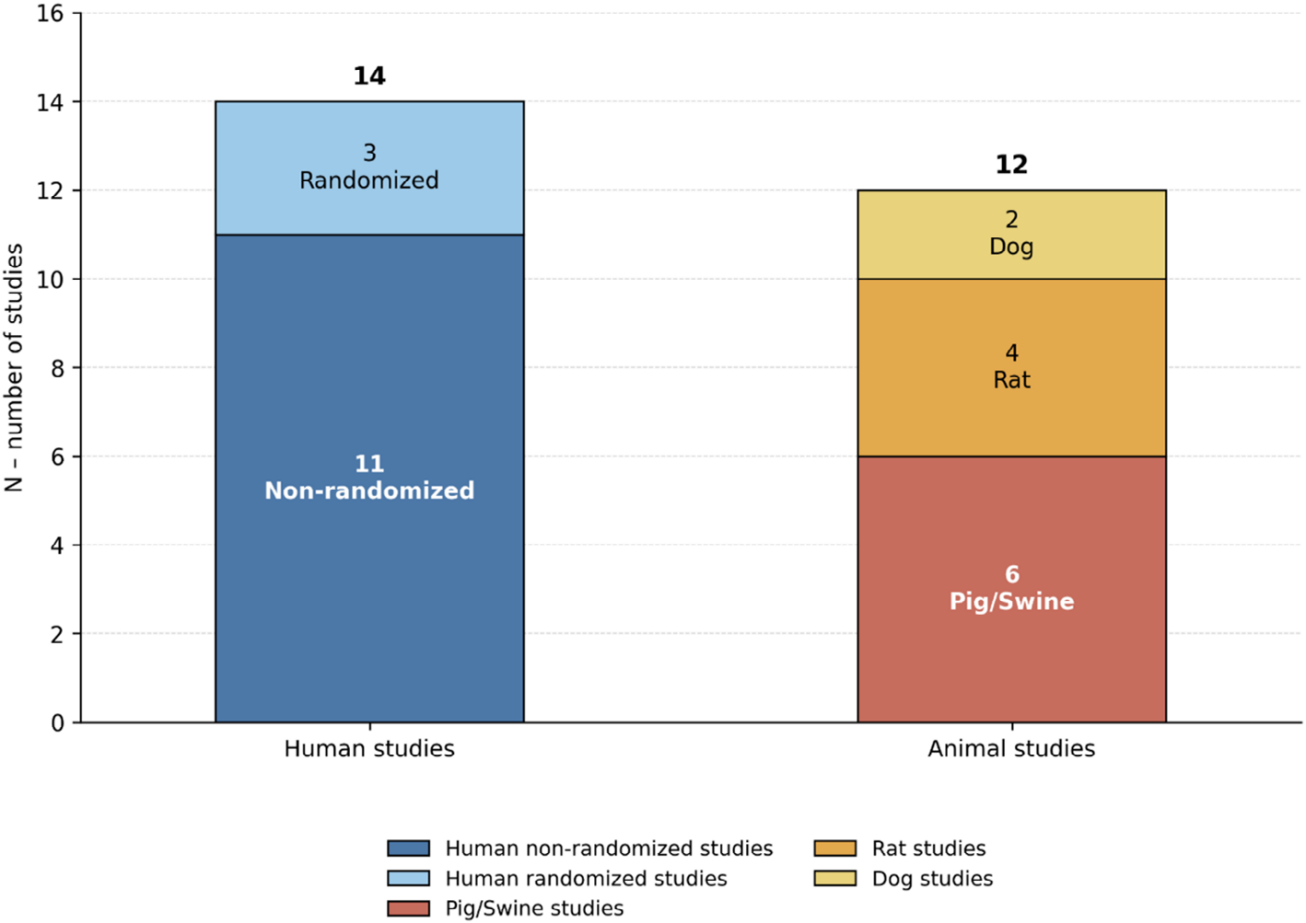
Distribution by cohort.

**FIGURE 3:**
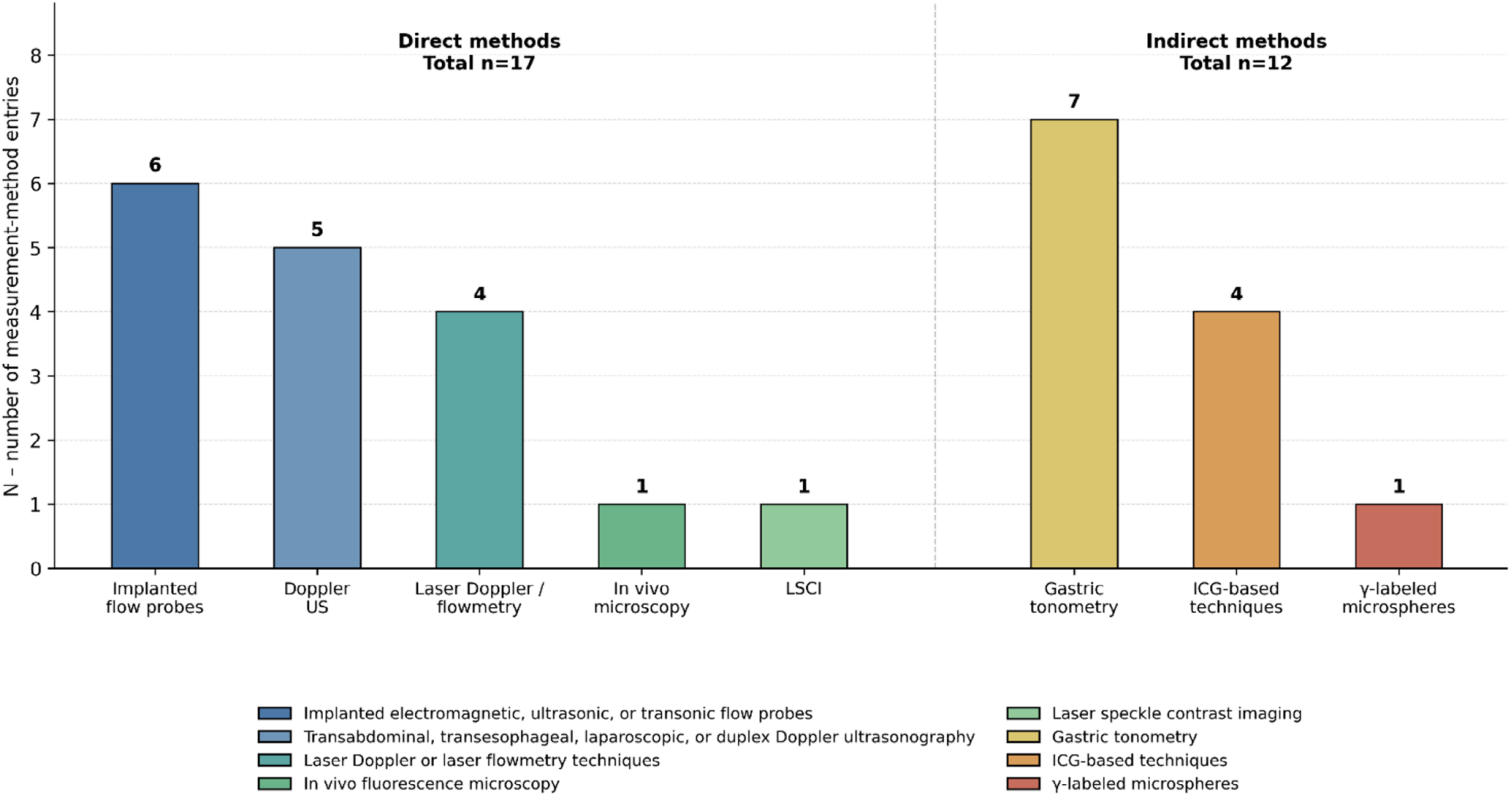
Measurement methods.

A detailed overview of study characteristics, interventions, measurement techniques, and main outcomes is presented in Table S1. The distribution of included studies by cohort is depicted in Figure 2, and the distribution of direct and indirect hepatosplanchnic perfusion measurement techniques is shown in Figure 3.

### 3.3 Critical appraisal of evidence

Formal study‐level risk‐of‐bias assessment was performed separately according to study design. Among the three randomized controlled human studies assessed with RoB 2, the overall risk of bias was lowest: two studies were rated as having low overall risk of bias, while one study, Reyad et al. (2013) raised some concerns due to potential bias in selection of the reported result. No randomized study was rated as having high risk of bias.

Among the 11 non‐randomized human studies assessed with ROBINS‐I, most studies showed moderate overall risk of bias. The most frequent concerns were related to confounding and selection of participants. One study, Thaler et al. (1996) was rated as having serious risk of bias due to serious concerns regarding confounding and participant selection, since the comparison groups were not randomly assigned and the open‐surgery group included patients with contraindications for laparoscopy.

Among the 12 experimental animal studies assessed with SYRCLE's risk‐of‐bias tool, the overall risk of bias was mostly unclear. This was mainly due to insufficient reporting of sequence generation, allocation concealment, random housing, and blinding procedures. One study, Ding et al. (2014) was rated as having high overall risk of bias because of incomplete outcome data. No animal study was rated as having unequivocally low overall risk of bias, although several studies had low risk in individual domains, particularly for baseline characteristics, incomplete outcome data, and selective outcome reporting.

**FIGURE 4:**
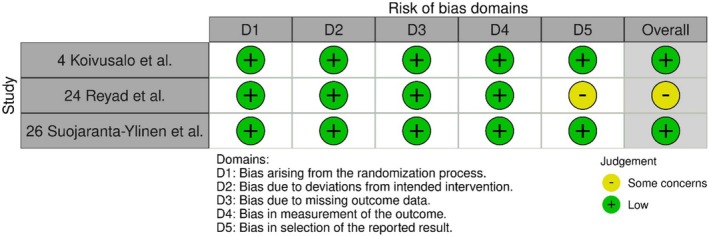
Risk‐of‐bias assessment of randomized human studies using RoB 2.

**FIGURE 5:**
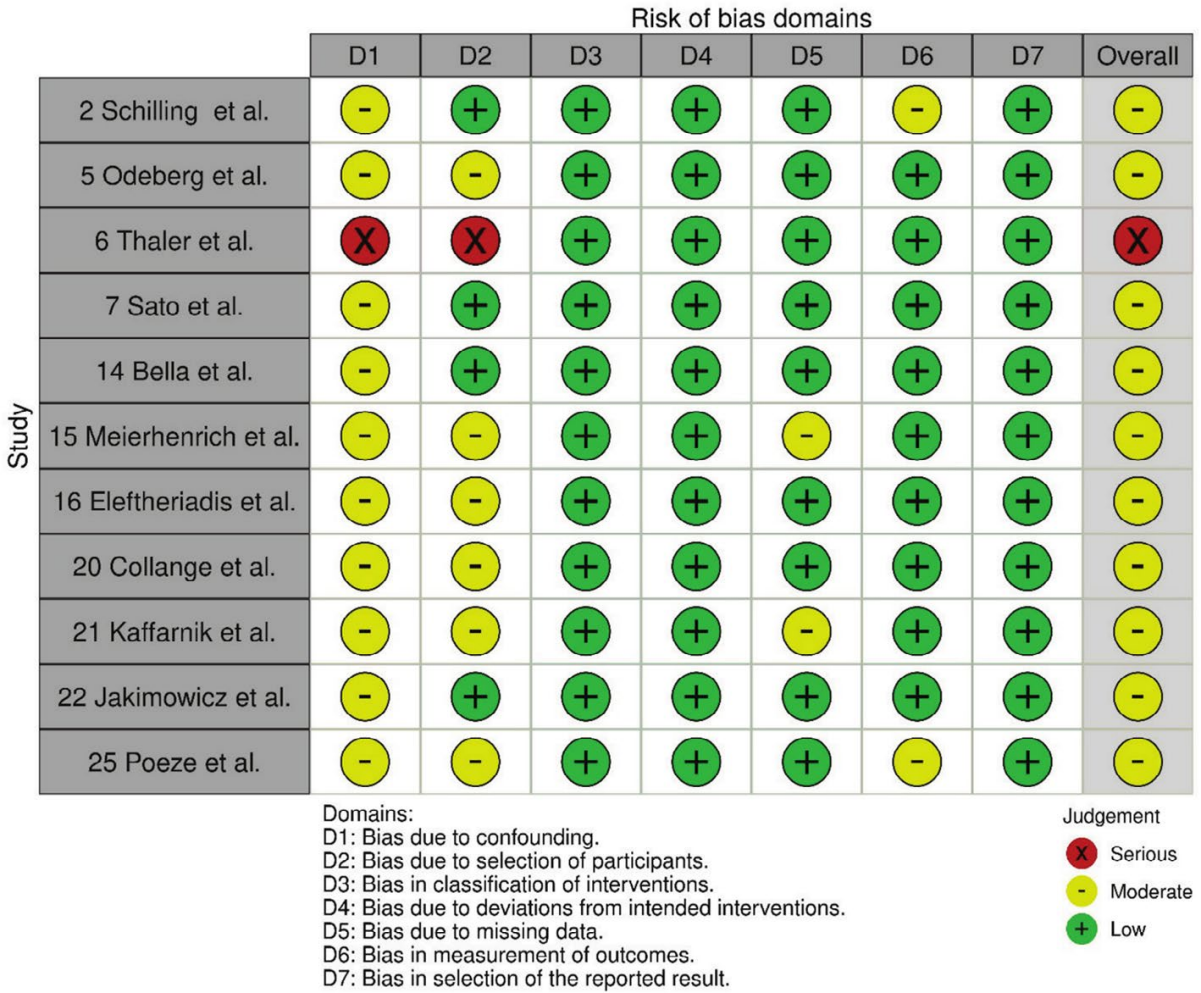
Risk‐of‐bias assessment of non‐randomized human studies using ROBINS‐I.

**FIGURE 6:**
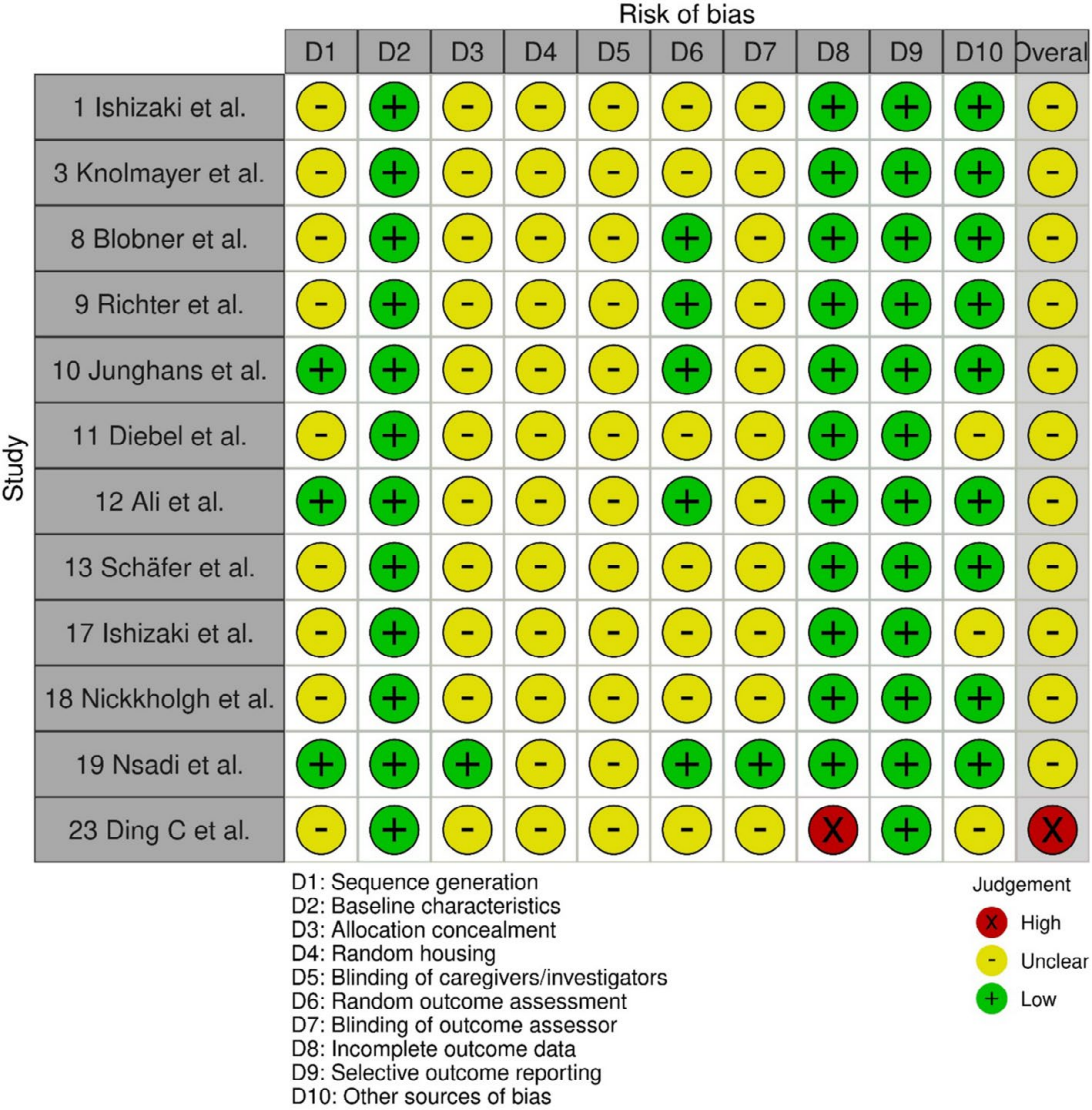
Risk‐of‐bias assessment of experimental animal studies using SYRCLE's risk‐of‐bias tool.

Overall, the conclusions of this systematic review are limited by predominantly moderate risk of bias in non‐randomized human studies and unclear risk of bias in most animal studies. The lowest risk of bias was observed among randomized human studies. The domain‐level and overall risk‐of‐bias judgments are summarized in Figures 4, 5, and 6.

### 3.4 Results of individual studies/ groups

Summaries of individual study outcomes are provided in Table S1.

**FIGURE 7:**
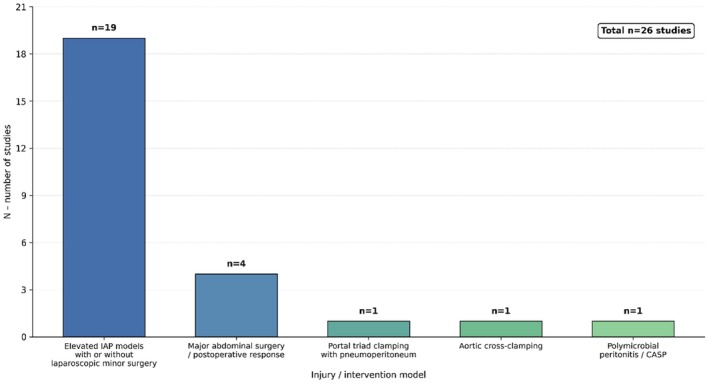
Distribution of included studies according to abdominal injury and intervention model.

The distribution of injury and intervention models is shown in Figure 7. Elevated intra‐abdominal pressure models, with or without laparoscopic minor surgery, represented the largest group of included studies (*n* = 19).

Four studies evaluated the postoperative hepatovisceral response after major abdominal surgery.

Portal triad clamping with pneumoperitoneum, aortic cross‐clamping, and polymicrobial peritonitis/CASP were each evaluated in one study.

#### 3.4.1 Pneumoperitoneum/IAP studies: Study characteristics and main perfusion findings

Among the 19 pneumoperitoneum/IAP studies, 10 were animal studies including 138 animals, and 9 were human studies including 297 participants. The mean sample size was 13.8 ± 6.1 (SD) animals per animal study and 33.0 ± 30.1 (SD) participants per human study. Overall, the studies included 435 subjects, with a mean sample size of 22.9 ± 22.8 (SD) per study.

The modes of IAP elevation included CO_2_ (Ali et al., 2005; Bella et al., 2004; Blobner et al., 1998; Eleftheriadis et al., 1996; Ishizaki et al., 1993a; Ishizaki et al., 1993b; Jakimowicz et al., 1998; Junghans et al., 1997; Knolmayer et al., 1998; Koivusalo et al., 1997; Meierhenrich et al., 2005; Nickkholgh et al., 2008; Nsadi et al., 2011; Odeberg et al., 1998; Richter et al., 2001; Sato et al., 2000; Schäfer et al., 2001; Schilling et al., 1997; Thaler et al., 1996), air (Blobner et al., 1998), argon (Junghans et al., 1997), helium (Junghans et al., 1997), warm Ringer's lactate (Diebel et al., 1992), and a CO_2_/ENO mixture (Ali et al., 2005).

Four studies reported no significant macrovascular or microvascular flow reduction at the investigated IAP levels (Bella et al., 2004; Nickkholgh et al., 2008; Odeberg et al., 1998; Thaler et al., 1996). However, Nickkholgh et al. (2008) showed preserved sinusoidal flow despite leukocyte and platelet adhesion, Kupffer‐cell activation, and biochemical liver injury after desufflation (Nickkholgh et al., 2008). Three additional studies reported changes that were either surrogate‐based or not fully quantifiable as pressure‐specific effect measures (Ishizaki et al., 1993a; Knolmayer et al., 1998; Koivusalo et al., 1997).

Among the remaining studies, the predominant finding was a decrease in portal venous blood flow (PVBF), total hepatic blood flow (THBF), hepatic venous blood flow, hepatic arterial flow, or microcirculatory perfusion with increasing IAP (Ali et al., 2005; Diebel et al., 1992; Eleftheriadis et al., 1996; Ishizaki et al., 1993b; Jakimowicz et al., 1998; Junghans et al., 1997; Richter et al., 2001; Sato et al., 2000; Schäfer et al., 2001; Schilling et al., 1997). Two studies showed a divergent response: Blobner et al. reported an initial increase in mesenteric arterial and portal venous flow at lower CO_2_‐IAP levels, followed by a decrease at higher pressures (Blobner et al., 1998), while Meierhenrich et al. reported increased right and middle hepatic venous flow during 12 mmHg CO_2_ pneumoperitoneum (Meierhenrich et al., 2005).

Additional important findings included the loss of the hepatic arterial buffer response (HABR) during progressive IAP elevation, as reported by Richter et al. (2001). Junghans et al. (1997) further demonstrated that body position and insufflation gas substantially influenced hepatic and portal flow, with the strongest impairment observed during head‐up positioning and argon insufflation. Conversely, Ali et al. reported that addition of ethyl nitrite (ENO) to CO_2_ attenuated the IAP‐associated reduction in liver blood flow, probably through NO‐mediated local and systemic vasodilation (Ali et al., 2005).

#### 3.4.2 Hepatosplanchnic vascular and perfusion response to major abdominal surgery

Kaffarnik et al. (2018) prospectively analyzed 25 patients undergoing major upper gastrointestinal surgery, including nine extended gastrectomies and 16 esophagectomies, and assessed hepatic circulation by duplex Doppler ultrasound preoperatively and on POD 1, 3, 5, and 10. Although the primary focus was postoperative liver function, the study demonstrated a marked postoperative hyperdynamic hepatic circulation: portal venous flow increased from 715 mL/min preoperatively to 1487.5 mL/min on POD 1 (+108%), then remained elevated at 1420.5 mL/min on POD 3 (+99%), 1177 mL/min on POD 5 (+65%), and 1050 mL/min on POD 10 (+47%). Common hepatic artery peak systolic velocity increased from 46 cm/s preoperatively to 85.5 cm/s on POD 1 (+86%) and 116 cm/s on POD 3 (+152%), before declining toward baseline at 93.5 cm/s on POD 5 (+103%) and 80 cm/s on POD 10 (+74%). Thus, major upper abdominal surgery was associated with a transient increase in both portal venous flow and hepatic arterial velocity, despite simultaneous postoperative impairment of liver metabolic function.

Reyad et al. (2013) included a control group of 20 patients undergoing pylorus‐preserving Whipple surgery without dobutamine. No direct macrovascular hepatosplanchnic flow was measured; splanchnic perfusion was assessed by gastric tonometry using PgCO_2_–PaCO_2_ gap and gastric pHi from baseline through 24 h after ICU admission. In controls, PgCO_2_–PaCO_2_ increased from ~3 mmHg to ~16–17 mmHg, while pHi decreased from ~7.35 to ~7.14–7.15, indicating microcirculatory gastric mucosal hypoperfusion that peaked around the end of dissection/anastomotic phase and persisted throughout the first 24 postoperative hours.

Poeze et al. (2002) showed that after major abdominal surgery, total hepatosplanchnic blood flow increased and gastric pHi decreased, suggesting postoperative macrovascular hyperperfusion with concomitant mucosal hypoperfusion/acidosis. This dissociation was more pronounced in patients who later developed hepatic dysfunction: they had significantly higher total hepatosplanchnic blood flow from 4 to 36 h postoperatively, significantly lower gastric pHi from 6 to 36 h, and reduced hepatic ICG extraction, with an approximately 20% reduction at 36 h.

Suojaranta‐Ylinen et al. (2000) studied 21 high‐risk patients undergoing major abdominal surgery, randomized to dopexamine or placebo. For the normal physiological response, only the placebo arm (*n* = 7) is relevant. Total splanchnic blood flow was measured by continuous ICG infusion after preoperative hemodynamic stabilization and again 6 h and 24 h after surgery. In the placebo group, splanchnic blood flow increased from 1.0 ± 0.2 (SD) L/min/m^2^ at baseline to 1.2 ± 0.2 (SD) L/min/m^2^ at both 6 h and 24 h, corresponding to an approximate 20% postoperative increase. Fractional splanchnic blood flow remained stable at 24%–27%, suggesting a proportional hyperdynamic splanchnic response rather than selective redistribution to the splanchnic bed. No microcirculatory variable such as gastric pHi or PCO_2_ gap was measured.

#### 3.4.3 Related experimental and clinical studies

Nsadi et al. (2011) and Collange et al. (2013) can be regarded as an extension of the pneumoperitoneum studies. Rather than examining the isolated effect of increased intra‐abdominal pressure, the authors used a combined portal triad clamping–pneumoperitoneum model to determine whether 15 mmHg IAP aggravates PTC‐induced hepatic ischemia. Compared with open PTC, laparoscopic PTC caused a significantly greater reduction in hepatic microcirculatory flow and tissue oxygen tension, with more severe hepatocellular injury after reperfusion, whereas pneumoperitoneum alone served as a negative control and did not significantly impair either parameter. Because the model involved deliberate vascular occlusion, the study was reported among the individual studies but excluded from the primary synthesis of the physiological response to isolated abdominal trauma.

Collange et al. (2013) and Kaffarnik et al. (2018) prospectively studied 18 patients undergoing elective infrarenal abdominal aortic aneurysm repair. Colonic mucosal perfusion was assessed by tonometric ΔPCO_2_ before clamping, at the end of clamping, and up to 24 h after reperfusion. ΔPCO_2_ increased from 1.8 to 2.6 kPa during clamping, decreased to 1.9 kPa by 3 h of reperfusion, showed a secondary peak of 2.7 kPa at 6 h, and normalized to 1.7 kPa by 24 h, indicating initial ischemic hypoperfusion followed by transient delayed reperfusion‐associated microcirculatory impairment. D‐lactate remained undetectable and did not reflect these changes.

Ding et al. (2014) evaluated 29 rats with colon ascendens stent peritonitis (CASP), a standardized model of polymicrobial abdominal sepsis created by inserting a stent into the ascending colon to induce continuous fecal leakage, and nine sham‐operated controls. Visceral microcirculation was assessed using laser speckle contrast imaging (LSCI), a non‐contact optical imaging technique that quantifies superficial tissue perfusion by analyzing speckle fluctuations generated by moving red blood cells. Measurements were performed at baseline and 4, 8, and 12 h after CASP induction. Ileal perfusion decreased by 21% at 4 h and 34% at 12 h, while renal cortical perfusion decreased by 23% at 8 h and 31% at 12 h. Hepatic perfusion declined by approximately 19% at 12 h but did not reach statistical significance, indicating that hepatic microcirculation was relatively preserved during early polymicrobial peritonitis compared with the intestinal and renal microcirculation.

### 3.5 Synthesis of results

#### 3.5.1 Methods used to assess hepatosplanchnic perfusion

Considerable methodological heterogeneity was observed across the included studies. Ultrasonography was the most frequently employed technique (*n* = 11). Six studies used implanted or intraoperative Doppler ultrasound probes for direct measurement of vascular blood flow, whereas five employed noninvasive or minimally invasive ultrasound techniques, including transabdominal duplex ultrasonography and transesophageal echocardiography. Gastric tonometry, an indirect method for assessing splanchnic mucosal perfusion through measurement of the mucosal–arterial CO_2_ gradient or intramucosal pH, was the second most commonly used technique (*n* = 7). Indocyanine green‐based methods, used to estimate total hepatosplanchnic or hepatic blood flow, and laser Doppler flowmetry, which assesses superficial tissue microcirculation, were each employed in four studies. Other less frequently used techniques included intravital fluorescence microscopy, laser speckle contrast imaging, and radiolabeled microspheres, each reported in a single study (Figure 3).

#### 3.5.2 Effect of pneumoperitoneum and intra‐abdominal pressure on hepatosplanchnic perfusion

**FIGURE 8:**
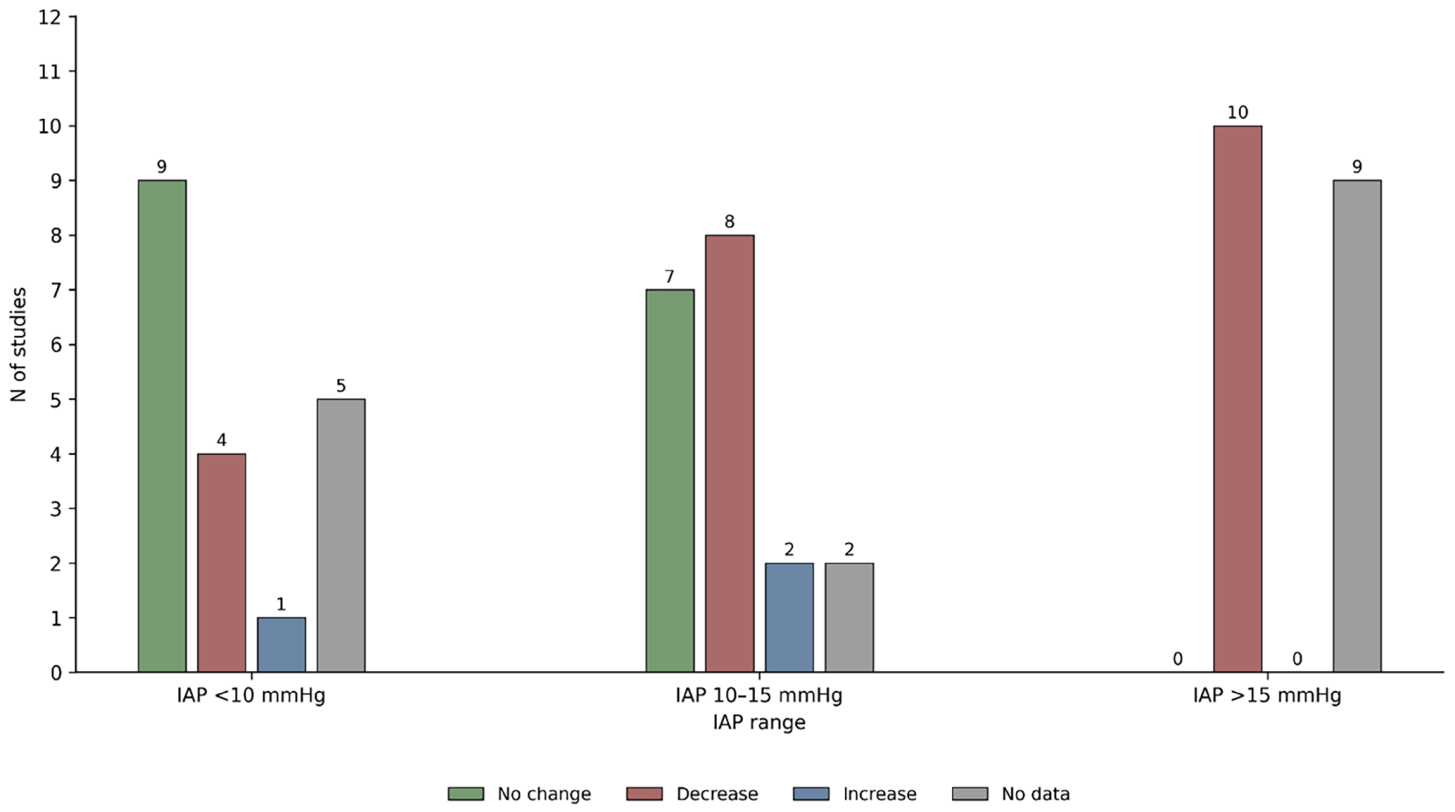
Direction of hepatosplanchnic perfusion changes across intra‐abdominal pressure categories.

Studies were classified according to whether they reported no change, a decrease, an increase, or no available data for hepatosplanchnic perfusion at IAP <10 mmHg, 10–15 mmHg, and > 15 mmHg. Bars represent the number of studies in each category. Pairwise statistical comparisons were performed using exact McNemar tests after dichotomizing outcomes as decrease versus non‐decrease and excluding studies without paired data for the respective comparison (Figure 8).

Among the 19 pneumoperitoneum/IAP studies, reported reductions in hepatosplanchnic perfusion became progressively more frequent with increasing intra‐abdominal pressure. A decrease was observed in 4 studies at IAP <10 mmHg, 8 studies at 10–15 mmHg, and 10 studies at >15 mmHg. Exact McNemar testing of studies with paired data showed no significant difference between <10 and 10–15 mmHg (*n* = 14, *p* = 0.250) or between 10–15 and > 15 mmHg (*n* = 8, *p* = 0.125). In contrast, decreases were significantly more frequent at >15 mmHg than at <10 mmHg (*n* = 8, *p* = 0.031). These findings support a pressure‐dependent increase in the likelihood of hepatosplanchnic perfusion impairment, with the clearest distinction between low‐ and high‐pressure pneumoperitoneum.

**FIGURE 9:**
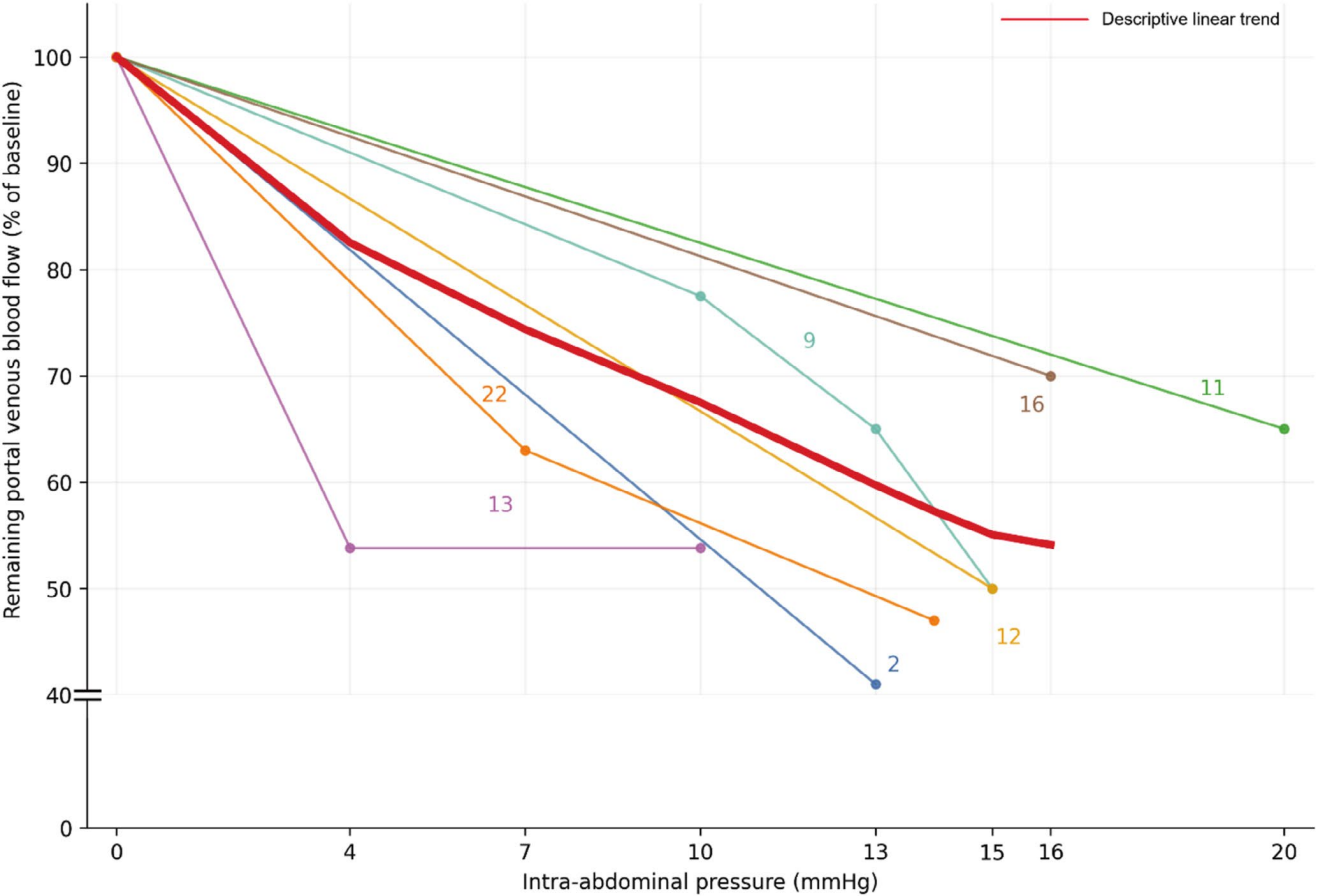
Descriptive pressure–response plot of portal venous blood flow during CO_2_ pneumoperitoneum.

To illustrate the relationship between increasing intra‐abdominal pressure and portal venous blood flow (PVBF) across the included studies, we constructed a descriptive plot using only studies that applied CO_2_ pneumoperitoneum and reported a clear numerical value or range for PVBF at a specified IAP. PVBF was expressed as the percentage of baseline flow remaining at each pressure level; where a range was reported, the midpoint was used. Values from each study were connected by straight lines and labeled with the corresponding reference number. The prominent red line represents a descriptive arithmetic mean across the plotted values and was included solely to facilitate visual interpretation of the overall trend. Across these studies, PVBF was generally preserved at baseline but progressively declined with increasing IAP, with substantial reductions already reported in some studies at 7–10 mmHg and more pronounced decreases at 13–16 mmHg. Thus, the figure visually supports a pressure‐dependent reduction in portal venous flow during CO_2_ pneumoperitoneum. Because of heterogeneity in study design, measurement technique, and reporting, this figure is intended only as an illustrative visualization and does not represent a formal statistical analysis or pooled effect estimate (Figure 9).

#### 3.5.3 Hepatosplanchnic vascular and perfusion response to major abdominal surgery

None of the four studies was primarily designed to characterize the normal physiological hepatosplanchnic response to major abdominal surgery. Kaffarnik et al. (2018) primarily assessed postoperative hepatic dysfunction using LiMAx, with hepatic blood‐flow measurements included to examine the influence of altered perfusion. Reyad et al. (2013) investigated whether intraoperative dobutamine improved gastric mucosal perfusion and postoperative outcome during Whipple surgery. Poeze et al. (2002) examined whether hepatosplanchnic inflammatory mediator production and perfusion changes preceded postoperative hepatic dysfunction, while Suojaranta‐Ylinen et al. (2000) evaluated the effects of dopexamine on regional blood flow, metabolism, and inflammation. Consequently, the unmodified surgical response was derived from secondary physiological outcomes in Kaffarnik and Poeze and from the untreated control or placebo groups in Reyad and Suojaranta‐Ylinen.

Despite differences in procedures and measurement techniques, the studies consistently indicated an early postoperative macrovascular hyperdynamic response. Kaffarnik et al. (2018) observed an increase in portal venous flow from 715 mL/min preoperatively to 1487.5 mL/min on POD 1 (+108%), followed by a gradual decline that nevertheless remained above baseline through POD 10. Common hepatic artery peak systolic velocity—not volumetric arterial flow—increased from 46 cm/s to 85.5 cm/s on POD 1 (+86%) and peaked at 116 cm/s on POD 3 (+152%) before declining toward baseline. In the placebo arm of Suojaranta‐Ylinen et al. (2000), ICG‐derived splanchnic blood flow increased from 1.0 ± 0.2 (SD) to 1.2 ± 0.2 (SD) L/min/m^2^ at both 6 and 24 h, whereas fractional splanchnic flow remained stable at 24%–27%, suggesting that the regional increase accompanied the systemic hyperdynamic response rather than selective redistribution to the splanchnic bed. Poeze et al. (2002) similarly documented increased ICG‐derived total hepatosplanchnic blood flow during the first 36 postoperative hours.

However, increased global flow did not ensure preserved regional microcirculation or hepatic function. In the untreated Whipple group studied by Reyad et al. (2013), the gastric mucosal–arterial PCO_2_ gap increased and gastric pHi decreased from the intraoperative period through the first 24 postoperative hours, indicating persistent gastric mucosal hypoperfusion despite the absence of direct macrovascular flow measurements. Poeze et al. (2002) demonstrated the same macro–microcirculatory dissociation: increased total hepatosplanchnic flow was accompanied by decreased gastric pHi, and patients who later developed hepatic dysfunction had higher calculated flow from 4 to 36 h, lower pHi from 6 to 36 h, and an approximately 20% reduction in hepatic ICG extraction at 36 h. Kaffarnik et al. (2018) likewise found postoperative hepatic hyperperfusion despite a simultaneous deterioration in enzymatic liver function.

Taken together, these studies suggest that major abdominal surgery produces an early systemic and hepatosplanchnic hyperdynamic macrovascular response, while gastric mucosal perfusion and hepatic metabolic capacity may remain impaired. The temporal pattern indicates that mucosal hypoperfusion may begin intraoperatively and persist for 24–36 h despite increased portal or total hepatosplanchnic flow. Direct quantitative comparison remains limited because the studies evaluated different physiological compartments: duplex Doppler measured portal volumetric flow but only hepatic arterial velocity, gastric tonometry provided an indirect regional measure of mucosal perfusion, and ICG‐derived flow may be difficult to interpret when hepatic extraction is impaired.

The overall evidence therefore supports a postoperative dissociation between macrovascular hepatosplanchnic blood flow and regional microcirculatory perfusion: portal and total hepatosplanchnic flow generally increased after major abdominal surgery, whereas gastric mucosal perfusion deteriorated and could remain impaired for 24–36 h.

#### 3.5.4 Related experimental and clinical studies

Nsadi et al. (2011), Collange et al. (2013), and Kaffarnik et al. (2018) examined perfusion changes caused by deliberate vascular occlusion. Nsadi et al. (2011) and Collange et al. (2013) showed that 15 mmHg CO_2_ pneumoperitoneum aggravated the reduction in hepatic microcirculatory flow and tissue oxygen tension during portal triad clamping. Collange et al. (2013) and Kaffarnik et al. (2018) demonstrated transient colonic mucosal hypoperfusion during infrarenal aortic cross‐clamping, with ΔPCO_2_ peaking at the end of clamping and normalizing within 24 h of reperfusion.

Ding et al. (2014) was the only study that directly investigated visceral perfusion after a primary intra‐abdominal injury. In 29 rats with colon ascendens stent peritonitis and nine sham‐operated controls, laser speckle contrast imaging was used to assess ileal, renal cortical, and hepatic microcirculation at baseline and 4, 8, and 12 h after injury. Ileal perfusion decreased by approximately 21% at 4 h and 34% at 12 h. Renal cortical perfusion remained initially preserved but declined significantly from 8 h onward, reaching an approximately 31% reduction at 12 h. Hepatic perfusion remained stable during the first 8 h and decreased by approximately 19% at 12 h, although this change was not statistically significant. No significant microcirculatory changes occurred in sham‐operated animals.

The principal finding from this limited evidence is that the early microcirculatory response to colonic injury is organ‐specific rather than uniform. Intestinal microvascular hypoperfusion developed first and progressed over time, renal impairment occurred later, and hepatic microcirculation remained comparatively preserved during the first 12 h. However, Ding et al. (2014) assessed only tissue‐level microvascular perfusion and did not measure portal, mesenteric, hepatic arterial, or total hepatosplanchnic blood flow. Consequently, the available literature provides no direct evidence on macrovascular hepatosplanchnic flow after isolated abdominal injury and only one experimental study describing its early organ‐specific microcirculatory response.

### 3.6 Reporting biases

Potential selective reporting within individual studies was assessed as part of RoB 2, ROBINS‐I, and SYRCLE. A separate synthesis‐level assessment of reporting bias, including publication bias and bias arising from unavailable studies or results, was not performed because the evidence was synthesized narratively in three methodologically distinct study‐design groups without quantitative pooling. Consequently, reporting bias across the available body of evidence cannot be excluded.

### 3.7 Certainty of evidence

No formal certainty‐of‐evidence assessment, such as GRADE, was performed and no categorical certainty rating was assigned. Confidence in the findings was nevertheless limited by heterogeneity in study designs, interventions, experimental models, measured vascular compartments, and perfusion‐assessment techniques. Additional limitations arose from the predominantly moderate risk of bias among non‐randomized human studies and the largely unclear risk of bias among animal studies. The randomized human studies had the most favorable risk‐of‐bias profile, but this evidence was based on only three methodologically distinct studies. Consequently, the findings should be interpreted as consistent physiological patterns rather than as precise or high‐certainty estimates of effect.

## 4 DISCUSSION

The principal finding of this review is the absence of studies specifically designed to characterize the unmodified hepatosplanchnic response to isolated abdominal organ injury. Although conceived as a scoping review, it was conducted within a systematic‐review framework, including an a priori protocol, systematic search, independent study selection, structured data extraction, design‐specific risk‐of‐bias assessment, and PRISMA‐based (Neudecker et al., 2002; Tricco et al., 2018) reporting.

None of the 26 included studies fully addressed the review question. Most investigated pneumoperitoneum, pharmacological optimization, postoperative physiological disturbances, or experimental vascular manipulation, with relevant perfusion data reported only as secondary outcomes or control observations. Ding et al. (2014) came closest by examining organ‐specific microcirculation after experimental colonic injury and peritonitis, but did not measure portal, mesenteric, hepatic arterial, or total hepatosplanchnic flow. The synthesis therefore represents an indirect reconstruction of the response to isolated abdominal injury rather than direct evidence from studies designed to define it.

The pneumoperitoneum/IAP studies constituted the largest and most internally coherent body of evidence. Most originated from the early period of widespread laparoscopic surgery: 12 of the 19 studies were published between 1992 and 1998, and many were designed to determine a physiologically acceptable working pressure for laparoscopy rather than to investigate abdominal injury. The present synthesis supports a pressure‐dependent increase in hepatosplanchnic perfusion impairment. A reduction was reported in four studies at IAP <10 mmHg, eight at 10–15 mmHg, and 10 at >15 mmHg. Paired comparisons demonstrated significantly more frequent reductions at >15 mmHg than at <10 mmHg, whereas differences between adjacent pressure categories were not statistically significant. These findings are consistent with individual studies reporting reductions in portal venous, mesenteric arterial, hepatic arterial, hepatic venous, total hepatic, or microvascular flow with increasing IAP (Ali et al., 2005; Diebel et al., 1992; Eleftheriadis et al., 1996; Ishizaki et al., 1993b; Jakimowicz et al., 1998; Junghans et al., 1997; Richter et al., 2001; Sato et al., 2000; Schäfer et al., 2001; Schilling et al., 1997). The descriptive PVBF plot further illustrated an inverse pressure–flow relationship during CO_2_ pneumoperitoneum, although it should be interpreted as a visualization of the overall reported pattern rather than as a pooled quantitative effect estimate.

The response at lower and intermediate pressures was less uniform. Some studies found no significant alteration in hepatic or splanchnic perfusion at the pressures examined (Bella et al., 2004; Nickkholgh et al., 2008; Odeberg et al., 1998; Thaler et al., 1996), whereas others detected reductions at pressures as low as 7–12 mmHg (Eleftheriadis et al., 1996; Jakimowicz et al., 1998; Sato et al., 2000). Blobner et al. (1998) observed an initial increase in portal venous and mesenteric arterial flow at lower CO_2_ pressures, followed by a decline at higher IAP, while Meierhenrich et al. (2005) reported increased hepatic venous flow at 12 mmHg. Some abnormalities persisted after desufflation or into the postoperative period (Ali et al., 2005; Ishizaki et al., 1993b; Koivusalo et al., 1997; Sato et al., 2000), with delayed recovery particularly evident in elderly patients (Sato et al., 2000). In contrast, Thaler et al. (1996) found no significant difference in gastric pHi between laparoscopic cholecystectomy at 15 mmHg and open surgery, Bella et al. (2004) reported no significant postoperative change in PVBF, and Nickkholgh et al. (2008) observed preserved sinusoidal perfusion despite evidence of endothelial activation and reperfusion injury.

Several studies also demonstrated that pressure alone did not determine the vascular response. Progressive IAP elevation abolished the hepatic arterial buffer response in a rat model (Richter et al., 2001), while body position and insufflation gas modified hepatic and portal flow, with greater impairment during head‐up positioning and with argon or helium than with CO_2_ (Junghans et al., 1997). Conventionally, pneumoperitoneum has been established at 12–15 mmHg (Raval et al., 2020). Current recommendations instead favor the lowest IAP that provides adequate operative exposure, with low‐pressure pneumoperitoneum generally defined as approximately 7–10 mmHg (Neudecker et al., 2002; Raval et al., 2020). Pressure should nevertheless be increased when visualization or operative conditions are inadequate. Taken together, the studies indicate a general pressure‐dependent deterioration in hepatosplanchnic perfusion, but their substantial heterogeneity in species, pressure levels, insufflation gas, body position, duration, measurement technique, and vascular compartment precludes a single universal safety threshold and supports narrative rather than quantitative synthesis.

The four major‐surgery studies were not designed primarily to characterize the normal postoperative vascular response; relevant findings were obtained from secondary physiological measurements or untreated control groups (Kaffarnik et al., 2018; Poeze et al., 2002; Reyad et al., 2013; Suojaranta‐Ylinen et al., 2000). Despite marked methodological differences, they consistently suggested an early postoperative hyperdynamic macrovascular response (Kaffarnik et al., 2018; Poeze et al., 2002; Suojaranta‐Ylinen et al., 2000). Portal venous flow approximately doubled on the first postoperative day after major upper gastrointestinal surgery (Kaffarnik et al., 2018), while ICG‐derived total splanchnic or hepatosplanchnic flow increased during the first 6–36 postoperative hours (Poeze et al., 2002; Suojaranta‐Ylinen et al., 2000). However, the macrovascular increase was not accompanied by uniform preservation of regional microvascular perfusion. Gastric tonometry demonstrated increasing mucosal–arterial PCO_2_ gradients and decreasing gastric pHi during Whipple and other major abdominal procedures, with abnormalities persisting for 24–36 h (Poeze et al., 2002; Reyad et al., 2013). The available evidence therefore indicates a macro–microcirculatory dissociation after major abdominal surgery: portal and total hepatosplanchnic blood flow increase during the early postoperative period, while gastric mucosal perfusion may simultaneously deteriorate (Kaffarnik et al., 2018; Poeze et al., 2002; Reyad et al., 2013; Suojaranta‐Ylinen et al., 2000). The magnitude of this dissociation cannot be directly quantified because the studies assessed different vascular compartments using non‐equivalent methods and postoperative time points (Kaffarnik et al., 2018; Poeze et al., 2002; Reyad et al., 2013; Suojaranta‐Ylinen et al., 2000).

Ding et al. (2014) provided the only direct evidence of visceral perfusion changes after a primary intra‐abdominal injury model. The response was organ‐specific and temporally sequential: ileal perfusion decreased by approximately 21% at 4 h and 34% at 12 h, renal cortical perfusion declined from 8 h onward to approximately 31% below baseline at 12 h, whereas hepatic perfusion remained relatively preserved, with only a non‐significant 19% reduction at 12 h. However, the study measured only tissue‐level perfusion and was judged to have a high overall risk of bias because of incomplete outcome data. Its findings should therefore be interpreted cautiously, and the corresponding portal and mesenteric macrovascular response remains unknown.

The hepatosplanchnic response to isolated abdominal organ injury remains poorly defined. Current evidence is derived mainly from pneumoperitoneum models, postoperative secondary outcomes, pharmacological control groups, and a single experimental peritonitis study rather than from studies specifically designed to examine isolated hollow‐ or parenchymal‐organ injury. No study has simultaneously assessed serial macrovascular and organ‐specific microvascular changes after such an injury.

Future research should therefore prospectively examine standardized isolated abdominal injuries, with elective abdominal resections serving as clinically relevant surrogates for traumatic injury. Portal, mesenteric, and hepatic arterial flow should be measured alongside organ‐level microcirculation from the immediate post‐injury or postoperative period through the following hours and days. Combining Doppler ultrasonography, ICG‐based global flow assessment, and regional microcirculatory techniques may distinguish local hypoperfusion, systemic hyperdynamic compensation, and delayed redistribution, provided that major confounders—including IAP, anesthesia, fluid therapy, vasoactive treatment, blood loss, systemic inflammation, and operative duration—are controlled. Without such purpose‐designed studies, the hepatosplanchnic response to isolated abdominal injury will remain inferred rather than directly established.

## 5 CONCLUSION

The available evidence is limited by substantial methodological heterogeneity and generally poor study quality. Most non‐randomized human studies had a moderate risk of bias, most animal studies had an unclear risk of bias, and one animal study had a high risk of bias. Only three randomized studies were available, and although their risk‐of‐bias profile was more favorable, they addressed different interventions and outcomes. Differences in species, injury models, measurement techniques, vascular compartments, and observation periods precluded quantitative pooling.

No included study was specifically designed to characterize the complete physiological hepatosplanchnic vascular response to isolated hollow‐ or parenchymal‐organ injury. The available evidence was derived mainly from pneumoperitoneum models, secondary postoperative measurements, untreated control groups, and a single experimental peritonitis model.

Despite these limitations, several consistent patterns emerged. Increasing intra‐abdominal pressure was associated with progressively more frequent reductions in portal venous, total hepatic, and microvascular perfusion, particularly above 15 mmHg, and higher pressures could impair compensatory mechanisms such as the hepatic arterial buffer response. After major abdominal surgery, portal and total hepatosplanchnic blood flow generally increased during the early postoperative period, while gastric mucosal perfusion could simultaneously deteriorate, indicating macro–microcirculatory dissociation. Experimental intra‐abdominal injury (bowel perforation) produced an organ‐specific response, with intestinal microcirculation affected earlier and more severely than renal or hepatic perfusion.

The hepatosplanchnic response to isolated abdominal injury therefore remains inferential rather than directly established. Prospective studies combining serial measurements of portal, mesenteric, and hepatic arterial flow with organ‐specific microcirculatory assessment are required to define its magnitude, timing, and physiological mechanisms.

## AUTHOR CONTRIBUTIONS

**Vid Pivec:** Conceptualization; data curation; formal analysis; funding acquisition; investigation; methodology; project administration; resources; validation; visualization. **Andrej Bergauer:** Formal analysis; investigation. **Anna Hawliczek:** Formal analysis; investigation. **Nandu Goswami:** Conceptualization; methodology; supervision.

## FUNDING INFORMATION

This research received no external funding. Publication fees were covered by the Medical University of Graz via publication funds allocated to the first author as part of the doctoral program.

## CONFLICT OF INTEREST STATEMENT

The authors declare that the research was conducted in the absence of any commercial or financial relationships that could be construed as a potential conflict of interest.

## ETHICS STATEMENT

This review was based exclusively on previously published data and did not involve the recruitment of human participants, the use of animals, or the collection of identifiable personal data. Therefore, ethics committee approval and informed consent were not required.

## Supporting information


Table S1.

Appendix S1.

Appendix S2.

## Data Availability

All data underlying this systematic review are included in the manuscript tables and figures. No additional datasets or code were generated.
