# Supplementary material for: Hepatosplanchnic perfusion changes following isolated abdominal trauma and surgery: A scoping review of evidence dominated by pneumoperitoneum models
Source: Physiol Rep. 2026 Aug 13;14(15):e71051. doi: 10.14814/phy2.71051 (PMC13470031; doi:10.14814/phy2.71051)
Supplement: Supplementary file 1 [file PHY2-14-e71051-s001.docx]

**List of Abbreviations (Alphabetically):**

**CASP –** Colon Ascendens Stent Peritonitis
**CO –** Cardiac Output **CO₂ –** Carbon Dioxide **CT –** Celiac Trunk **CVP –** Central Venous Pressure
**ENO –** Ethyl Nitrite **GRADE –** Grading of Recommendations Assessment, Development and Evaluation **HA –** Hepatic Artery **HABF –** Hepatic Arterial Blood Flow **HABR –** Hepatic Arterial Buffer Response **HAF –** Hepatic Arterial Flow **HBF –** Hepatic Blood Flow **HD− –** Absence of Hepatic Dysfunction **HD+ –** Presence of Hepatic Dysfunction **IAP –** Intra-Abdominal Pressure **ICG –** Indocyanine Green **ICG-PDR –** Indocyanine Green Plasma Disappearance Rate **ICU –** Intensive Care Unit **IVC –** Inferior Vena Cava **LC –** Laparoscopic Cholecystectomy **LiMAx –** Liver Maximum Capacity Test **LSCI –** Laser Speckle Contrast Imaging **MAP –** Mean Arterial Pressure **MEDLINE –** Medical Literature Analysis and Retrieval System Online **MeSH –** Medical Subject Headings **MHVBF –** Middle Hepatic Vein Blood Flow **MHVBFI –** Middle Hepatic Vein Blood Flow Index **NAG –** N-Acetyl-β-D-Glucosaminidase **NO –** Nitric Oxide **NS –** Not Statistically Significant **O₂ –** Oxygen **PaCO₂ –** Arterial Partial Pressure of Carbon Dioxide **PCO₂ –** Partial Pressure of Carbon Dioxide **PgCO₂ –** Gastric Mucosal Partial Pressure of Carbon Dioxide **pHi –** Gastric Intramucosal pH **POD –** Postoperative Day **PRISMA –** Preferred Reporting Items for Systematic Reviews and Meta-Analyses **PTC –** Portal Triad Clamping **PV –** Portal Vein **PVBF –** Portal Venous Blood Flow **RBF –** Renal Blood Flow **RBC –** Red Blood Cell **RHVBFI –** Right Hepatic Vein Blood Flow Index **RoB –** Risk of Bias **RoB 2 –** Cochrane Risk of Bias 2 Tool
**ROBINS-I** – Risk Of Bias In Non-randomized Studies of Interventions **SMA –** Superior Mesenteric Artery **SMABF –** Superior Mesenteric Arterial Blood Flow **SVR –** Systemic Vascular Resistance **SYRCLE –** Systematic Review Centre for Laboratory Animal Experimentation **TEE –** Transesophageal Echocardiography **THBF –** Total Hepatic Blood Flow **TLBF –** Total Liver Blood Flow **TNF-α –** Tumor Necrosis Factor Alpha **US –** Ultrasonography **ΔPCO₂ –** Mucosal–Arterial Carbon Dioxide Partial-Pressure Gradient
