# Supplementary material for: Hepatosplanchnic perfusion changes following isolated abdominal trauma and surgery: A scoping review of evidence dominated by pneumoperitoneum models
Source: Physiol Rep. 2026 Aug 13;14(15):e71051. doi: 10.14814/phy2.71051 (PMC13470031; doi:10.14814/phy2.71051)
Supplement: Supplementary file 3 [file PHY2-14-e71051-s003.docx]

| **Ref.** | **Author** | **Year** | **Study design** | **Model** | **Experimental setting** | **Measuring device** | **Type of intervention** | **N** | **Study duration / measurement points** | **Main macrovascular outcomes** | **Main microvascular outcomes** | **Magnitude of effect** | **Main physiological finding** | **RoB tool** | **Overall RoB** |
| --- | --- | --- | --- | --- | --- | --- | --- | --- | --- | --- | --- | --- | --- | --- | --- |
| **1** | Ishizaki Y et al. | 1993 | Experimental controlled study | Animal (dogs) | Systemic and visceral hemodynamic monitoring during pneumoperitoneum | Electromagnetic flow probes | CO₂ pneumoperitoneum (8, 12 and 16 mmHg IAP) | 21 (3×7) | Baseline; 1, 2 and 3 h | ↓ PVBF, ↓ SMABF, ↓ THBF at 16 mmHg | None | Significant reduction only at **16 mmHg**; no significant changes at **8–12 mmHg** | Pressure threshold for macrovascular impairment at **16 mmHg**; 8–12 mmHg preserved hepatosplanchnic perfusion. | SYRCLE | Uncelar |
| **2** | Schilling MK et al. | 1997 | Prospective observational study | Human | Visceral microcirculatory monitoring during laparoscopic surgery | Custom-made laser Doppler flow probe | CO₂ pneumoperitoneum (0, 10 and 15 mmHg IAP) during laparoscopic appendectomy/cholecystectomy | 18 | Measurements at 0, 10 and 15 mmHg | Not directly measured | Gastric, duodenal, jejunal, colonic, hepatic and peritoneal perfusion | Stomach ↓40–54%; Jejunum ↓32%; Colon ↓44%; Liver ↓39%; Peritoneum ↓60%; Duodenum ↓11% | Marked microcirculatory impairment develops between **10 and 15 mmHg** despite preserved systemic hemodynamics. | ROBINS-I* | Moderate |
| **3** | Knolmayer TJ et al. | 1998 | Prospective controlled study | Animal (swine) | Gastric perfusion during graded pneumoperitoneum | Gastric tonometry (gastric intramucosal pH) | CO₂ pneumoperitoneum (0–18 mmHg) | 16 (9 intervention, 7 controls) | Every 30 min during 3 h | Systemic hemodynamics unchanged | ↓ Gastric mucosal perfusion (↓ pHi) | Significant pHi decrease at **16 and 18 mmHg**; no significant changes at constant **8 mmHg** | Microcirculatory impairment precedes detectable systemic hemodynamic changes. | SYRCLE | Unclear |
| **4** | Koivusalo AM et al. | 1997 | Prospective randomized controlled trial | Human | Systemic and splanchnic monitoring during laparoscopic cholecystectomy comparing CO₂ pneumoperitoneum with gasless abdominal wall lift | Gastric tonometry (TRIP®/Tonocap®), arterial blood gases, MAP, urinary NAG | CO₂ pneumoperitoneum (12–13 mmHg) vs gasless abdominal wall lift | 30 (15/15; 1 excluded) | Baseline; 30 & 60 min intra-op; 0, 60 & 180 min postop | ↑ MAP with CO₂ pneumoperitoneum | ↓ Gastric mucosal pHi; ↑ gastric PCO₂; ↓ urine output; ↑ urinary NAG | MAP ↑ (P<0.001); pHi remained significantly lower for 3 h (P<0.001) | Elimination of pneumoperitoneum largely prevents the splanchnic and renal perfusion disturbances associated with laparoscopic surgery. | RoB 2 | Low |
| **5** | Odeberg S et al. | 1998 | Prospective observational physiological study | Human | Hepatosplanchnic hemodynamic monitoring during laparoscopic cholecystectomy | Indocyanine green (ICG) constant infusion technique | CO₂ pneumoperitoneum (10–13 mmHg IAP) during laparoscopic cholecystectomy | 5 | Baseline and 60 min | Hepatic blood flow (HBF), systemic hemodynamics, hepatosplanchnic O₂ transport | None | No significant change in HBF, hepatosplanchnic oxygen consumption or oxygen extraction | CO₂ pneumoperitoneum of **10–13 mmHg** did not measurably impair global hepatosplanchnic perfusion. | ROBINS-I* | Moderate |
| **6** | Thaler W et al. | 1996 | Prospective comparative study | Human | Visceral perfusion monitoring during laparoscopic versus open cholecystectomy | Gastric tonometry (gastric intramucosal pH, pHi) | Laparoscopic cholecystectomy with 15 mmHg CO₂ pneumoperitoneum vs open cholecystectomy | 63 (31 laparoscopic, 32 open) | Surgery + 4 h postoperative | No significant differences in systemic hemodynamics between groups | Gastric mucosal pHi | No significant differences in gastric pHi throughout the study | At an IAP of 15 mmHg, laparoscopic cholecystectomy did not impair gastric mucosal perfusion more than open cholecystectomy. | ROBINS-I | Serious |
| **7** | Sato K et al. | 2000 | Prospective comparative study | Human | Visceral hemodynamic monitoring during laparoscopic and open cholecystectomy | Transesophageal Doppler ultrasound measuring middle hepatic vein blood flow (MHVBF) | CO₂ pneumoperitoneum (9–12 mmHg) during laparoscopic cholecystectomy (adult and elderly) vs open cholecystectomy | 100 (49 adult LC, 36 elderly LC, 15 elderly open) | Surgery + 30 min after deflation | Middle hepatic vein blood flow (MHVBF) | None | Significant intraoperative reduction of MHVBF; recovery significantly delayed in elderly patients | Even relatively low-pressure pneumoperitoneum reduces hepatic venous blood flow, with slower recovery in elderly patients. | ROBINS-I | Moderate |
| **8** | Blobner M et al. | 1998 | Prospective controlled experimental study | Pig | Systemic and visceral hemodynamic monitoring during graded CO₂ versus air pneumoperitoneum | Surgically implanted transit-time ultrasonic flow probes (IVC, PV, SMA) and cardiac output monitoring | Stepwise IAP increase (4 mmHg increments to 24 mmHg) using CO₂ or air | 24 | Stepwise insufflation + 30 min | Cardiac output, IVC flow, PVBF, SMABF | None | Air: progressive reduction of PVBF and SMABF. CO₂: initial increase followed by reduction above 12 mmHg. Cardiac output decreased with increasing IAP. | The type of insufflation gas independently influences the macrovascular response. CO₂ produces an initial vasodilatory response not seen with air, whereas higher IAP (>12 mmHg) impairs hepatosplanchnic perfusion regardless of gas. | SYRCLE | Unclear |
| **9** | Richter S et al. | 2001 | Prospective controlled experimental study | Rat | Visceral hemodynamic monitoring during graded CO₂ pneumoperitoneum compared with graded portal vein stenosis | Surgically implanted ultrasonic flow probes measuring portal vein and hepatic artery blood flow | CO₂ pneumoperitoneum (2 mmHg increments to 18 mmHg, maintained for 2 h) vs graded portal vein occlusion | 14 (7 CO₂, 7 PV stenosis) | Stepwise insufflation + 2 h | Portal venous blood flow (PVBF), hepatic arterial blood flow (HABF), hepatic arterial buffer response (HABR) | None | Progressive reductions in PVBF and HABF during CO₂ pneumoperitoneum with complete loss of HABR. PV stenosis preserved HABR and compensated for up to 50% PVBF reduction. | CO₂ pneumoperitoneum abolishes the hepatic arterial buffer response, whereas isolated portal vein stenosis preserves physiological hepatic autoregulation. | SYRCLE | Unclear |
| **10** | Junghans T et al. | 1997 | Prospective controlled experimental study | Pig | Systemic and visceral hemodynamic monitoring during pneumoperitoneum using different gases, body positions and IAP levels | Surgically implanted transonic perivascular flow probes on hepatic artery, portal vein and renal artery; Swan–Ganz catheter | CO₂, helium or argon pneumoperitoneum at 8, 12 and 16 mmHg in supine, head-up and head-down positions | 18 (6 CO₂, 6 helium, 6 argon) | Nine pressure/position combinations; 15-min adaptation + 15-min recording each (≈3 h) | Total liver blood flow (TLBF), hepatic arterial flow (HAF), portal venous blood flow (PVBF), renal blood flow (RBF), cardiac output (CO), systemic vascular resistance (SVR), central venous pressure (CVP) | None | TLBF decreased with increasing IAP, head-up position and all gases. Argon produced the greatest reduction (TLBF to 74% of baseline at 16 mmHg). PVBF depended mainly on gas type and body position, only minimally on pressure. Systemic hemodynamics did not correlate with hepatic blood flow. | Hepatic perfusion is independently influenced by intra-abdominal pressure, body position and insufflation gas. Argon causes the greatest reduction in liver perfusion, whereas CO₂ and helium are less detrimental. Systemic hemodynamics are poor predictors of hepatosplanchnic perfusion. | SYRCLE | Unclear |
| **11** | Diebel LN et al. | 1992 | Prospective controlled experimental study | Pig | Visceral hemodynamic monitoring during graded intra-abdominal instillation of warm (37°C) Ringer lactate | Surgically implanted transonic flow probes (portal vein, hepatic artery); hepatic microcirculatory flow assessment | Warm Ringer lactate instillation producing IAP of 10, 20, 30 and 40 mmHg | 5 | Baseline + 15 min at each pressure level | Portal venous blood flow (PVBF), hepatic arterial flow (HAF), total hepatic blood flow | Hepatic microcirculatory blood flow | Progressive IAP-dependent reduction of PVBF, HAF and hepatic microcirculatory perfusion | Even modest elevations of IAP profoundly impair both hepatic macrovascular and microvascular perfusion. | SYRCLE | Unclear |
| **12** | Ali NA et al. | 2005 | Prospective randomized controlled experimental study | Pig | Hepatosplanchnic perfusion during CO₂ pneumoperitoneum with or without ethyl nitrite (ENO) | Laser Doppler flow probes on liver and kidney surfaces | 15 mmHg CO₂ pneumoperitoneum vs 15 mmHg CO₂ + 100 ppm ENO | 12 (6+6; final analysis n=11) | 40 min baseline, 60 min insufflation, 60 min after desufflation | No direct macrovascular flow measurements | Hepatic and renal microvascular blood flow | CO₂ reduced hepatic microvascular blood flow by ≈50%, persisting after desufflation. ENO significantly attenuated both acute and persistent reductions. Kidney perfusion was unchanged. | CO₂ pneumoperitoneum induces prolonged hepatic microvascular hypoperfusion that can be partially prevented by increasing nitric oxide bioavailability. | SYRCLE | Unclear |
| **13** | Schäfer M et al. | 2001 | Prospective controlled experimental study | Rat | Systemic and hepatosplanchnic circulation during CO₂ pneumoperitoneum | γ-labeled microsphere technique | CO₂ pneumoperitoneum at 4 and 10 mmHg | 12 | 30 min baseline → 30 min at 4 mmHg → 30 min at 10 mmHg → 2 min after desufflation | Cardiac output, portal venous flow, hepatic arterial flow, total hepatic blood flow | Organ perfusion of liver, spleen, pancreas, kidneys, stomach, small bowel and colon | Portal venous flow fell to approximately 54% of baseline; liver perfusion decreased to 63–71%, spleen 38–65%, pancreas 51–58%, kidneys 35–41%; hollow organs were less affected. | Solid abdominal organs are considerably more susceptible than hollow viscera to pneumoperitoneum-induced hypoperfusion. Recovery of systemic hemodynamics precedes recovery of regional splanchnic perfusion. | SYRCLE | Unclear |
| **14** | Bella M et al. | 2004 | Prospective observational physiological study | Human | Portal venous hemodynamic monitoring before and after laparoscopic cholecystectomy | Transabdominal Doppler ultrasonography | Elective laparoscopic cholecystectomy | 30 | Preoperative, 24 h, 48 h | Portal vein diameter, flow velocity and portal venous blood flow (PVBF) | None | Mean PVBF 725 ± 187 ml/min before surgery; no significant changes at 24 h or 48 h postoperatively | Portal venous blood flow remains essentially unchanged during the first 48 postoperative hours after uncomplicated laparoscopic cholecystectomy. | ROBINS-I | Moderate |
| **15** | Meierhenrich R et al. | 2005 | Prospective controlled physiological study | Human | Hepatic blood flow monitoring during laparoscopic surgery | Multiplane transesophageal echocardiography (TEE) with Doppler of right and middle hepatic veins | 12 mmHg CO₂ pneumoperitoneum during laparoscopic cholecystectomy and laparoscopic inguinal hernia repair | 24 (12+12; 9 additional patients excluded because adequate TEE imaging was unavailable) | Baseline, 5, 10, 20, 30, 40 min, 1 and 5 min after desufflation | Right hepatic vein blood flow index (RHVBFI), middle hepatic vein blood flow index (MHVBFI) | None | RHV blood flow increased significantly throughout pneumoperitoneum (e.g. 228 → 616 ml/min/m² during laparoscopic cholecystectomy); MHV flow also increased. Values returned rapidly to baseline after desufflation. | Low-pressure (12 mmHg) CO₂ pneumoperitoneum increases hepatic venous blood flow in healthy adults, suggesting that CO₂-induced local vasodilation may outweigh the mechanical effects of increased IAP at lower pressures. | ROBINS-I | Moderate |
| **16** | Eleftheriadis E et al. | 1996 | Prospective comparative physiological study | Human | Intraoperative assessment of hepatosplanchnic microcirculation during laparoscopic versus open cholecystectomy | Intraparenchymal laser Doppler microprobe and gastric tonometry | Laparoscopic cholecystectomy (CO₂ <12 mmHg) vs open cholecystectomy | 16 (8+8) | Continuous intraoperative measurements; after desufflation | None | Hepatic microcirculation; gastric intramucosal pH | Hepatic perfusion decreased from 57.5 ±18.1 to 22.2 ±5.5 PU; gastric pHi fell to 7.15 ±0.16; both normalized after desufflation | CO₂ pneumoperitoneum induces marked but rapidly reversible hepatosplanchnic microcirculatory hypoperfusion accompanied by gastric mucosal acidosis. | ROBINS-I | Moderate |
| **17** | Ishizaki Y et al. | 1993 | Prospective controlled experimental study | Dog | Systemic and hepatosplanchnic hemodynamic monitoring during prolonged CO₂ pneumoperitoneum | Transit-time ultrasonic flow probes (PV, HA, SMA) and cardiac output monitoring | 16 mmHg CO₂ pneumoperitoneum for 3 h followed by decompression | 6 | Baseline; 30 min; 1, 2, 3 h; 30 min after decompression | Cardiac output, SMA flow, PVBF, HAF, TLBF | None | Progressive reductions in PVBF, SMA flow and TLBF throughout pneumoperitoneum; HAF relatively preserved; recovery after decompression | Prolonged high-pressure pneumoperitoneum progressively impairs hepatosplanchnic perfusion, primarily through portal and mesenteric flow reduction, with only partial hepatic arterial compensation. | SYRCLE | Unclear |
| **18** | Nickkholgh A et al. | 2007 | Prospective controlled experimental study | Rat | Hepatic microcirculation after CO₂ pneumoperitoneum and reperfusion | In vivo fluorescence intravital microscopy | 90 min CO₂ pneumoperitoneum at 8 or 12 mmHg followed by desufflation | 8–10/group | Measurements during reperfusion after 90-min pneumoperitoneum | None | Functional sinusoidal density, RBC velocity, leukocyte rolling and adhesion, platelet adhesion | Sinusoidal perfusion and RBC velocity unchanged; significant increases in leukocyte and platelet adhesion, more pronounced at 12 mmHg | Microvascular blood flow rapidly recovers after desufflation, but endothelial activation persists, demonstrating a hepatic ischemia–reperfusion response despite restored perfusion. | SYRCLE | Unclear |
| **19** | Nsadi B et al. | 2011 | Prospective controlled experimental study | Swine | Hepatic ischemia during laparoscopic versus open portal triad clamping | Clark-type oxygen electrode and laser Doppler flowmetry | 15 mmHg CO₂ pneumoperitoneum with portal triad clamping vs open portal triad clamping | 15 | Continuous measurements during ischemia and reperfusion (~4 h) | None | Hepatic microcirculation; liver tissue oxygen tension | Laparoscopic PTC produced a more rapid and more severe decrease in hepatic microcirculation and oxygenation than open PTC; delayed reperfusion recovery | Pneumoperitoneum substantially aggravates hepatic ischemia during portal triad clamping, likely by reducing hepatic venous backflow. | SYRCLE | Unclear |
| **20** | Collange O et al. | 2013 | Prospective observational physiological pilot study | Human | Intestinal ischemia-reperfusion during elective infrarenal abdominal aortic aneurysm repair | TRIP gastric tonometer, TNF-α and D-lactate assays | Infrarenal aortic cross-clamping followed by reperfusion | 18 | Baseline; during clamping; after reperfusion; up to 24 h | None | Gastric mucosal ΔPCO₂, TNF-α, D-lactate | Significant increase in ΔPCO₂ during clamping and reperfusion; TNF-α showed similar kinetics; D-lactate remained undetectable | Temporary interruption of splanchnic inflow induces transient intestinal microcirculatory ischemia followed by reperfusion-associated inflammatory activation despite no biochemical evidence of intestinal necrosis. | ROBINS-I | Moderate |
| **21** | Kaffarnik M et al. | 2018 | Prospective observational physiological study | Human | Major upper GI surgery (16 esophagectomies, 9 extended gastrectomies); open vs minimally invasive | Duplex Doppler US, ICG-PDR, LiMAx® | Major upper abdominal surgery | 55 enrolled; 25 analyzed; 18 at POD10 | Baseline; POD1, POD3, POD5, POD10 | Portal vein flow, hepatic artery velocity, ICG-PDR | None | Portal flow approximately doubled on POD1; hepatic artery velocity increased 2–2.5-fold, peaking on POD3; ICG-PDR increased (~21→26 %/min); LiMAx decreased (413→290 μg/kg/h) despite increased flow | Major upper abdominal surgery induces transient hepatovisceral hyperperfusion despite temporary impairment of metabolic liver function, indicating dissociation between perfusion and hepatic functional capacity. | ROBINS-I | Moderate |
| **22** | Jakimowicz J et al. | 1998 | Prospective physiological observational study | Human | Elective laparoscopic cholecystectomy | Laparoscopic duplex Doppler ultrasonography | CO₂ pneumoperitoneum (0, 7 and 14 mmHg) | 11 | Sequential measurements at 0, 7 and 14 mmHg; after desufflation | Portal venous blood flow | None | PV flow: 990→568 mL/min (-37%) at 7 mmHg; 440 mL/min (-53%) at 14 mmHg; complete recovery after desufflation | Portal venous blood flow decreases in a pressure-dependent manner during pneumoperitoneum and rapidly normalizes after decompression, indicating a reversible mechanical effect of increased IAP. | ROBINS-I | Moderate |
| **23** | Ding C et al. | 2014 | Prospective controlled experimental study | Rat | Colon ascendens stent peritonitis (CASP) | Laser speckle contrast imaging (LSCI) | Polymicrobial peritonitis | 29 septic + 9 sham | Baseline; 4 h, 8 h, 12 h | None | Ileal, renal cortical and hepatic microvascular perfusion | Ileal perfusion decreased 21% by 4 h and 34% by 12 h; renal perfusion decreased 31% by 12 h; hepatic perfusion decreased ~19% (NS) | Peritonitis induces sequential organ-specific microcirculatory impairment (gut > kidney > liver), demonstrating that systemic stability does not exclude regional microvascular failure. | SYRCLE | High |
| **24** | Reyad AR et al. | 2013 | Prospective randomized controlled trial | Human | Pylorus-preserving Whipple procedure | Gastric tonometry (PgCO₂−PaCO₂ gap, pHi) | Whipple surgery ± dobutamine | 60 (20/group) | Baseline; intraoperatively; ICU; 3, 6, 12 and 24 h postop | No direct macrovascular measurements | Gastric mucosal perfusion (PCO₂ gap, pHi) | PCO₂ gap increased from ~3 to 16–17 mmHg (+430–470%); pHi decreased 7.35→7.14; abnormalities persisted at 24 h; dobutamine partially attenuated changes | Major upper abdominal surgery induces marked and persistent gastric mucosal hypoperfusion despite stable systemic hemodynamics. No conclusions regarding global hepatovisceral blood flow can be drawn. | RoB 2 | Some concerns |
| **25** | Poeze M et al. | 2002 | Retrospective analysis of a randomized clinical trial | Human | Major elective high-risk abdominal surgery | Continuous ICG infusion (Fick principle), gastric tonometry | Major abdominal surgery (heterogeneous procedures) | 26 (20 HD−, 6 HD+) | Baseline; 4 h, 24 h, 36 h | Total hepatovisceral blood flow (ICG-derived) | Gastric intramucosal pH | HD−: hepatovisceral flow increased ~0.85→1.20 L/min/m² (+38–41%); pHi decreased 7.35→7.28 with partial recovery. HD+: higher calculated flow but reduced ICG extraction | Major abdominal surgery induces moderate hepatovisceral hyperperfusion while gastric mucosal perfusion transiently deteriorates, demonstrating dissociation between macrovascular flow and tissue perfusion. Interpretation of ICG-derived flow is limited in hepatic dysfunction. | ROBINS-I | Moderate |
| **26** | Suojaranta-Ylinen RT et al. | 2000 | Prospective randomized controlled trial | Human | Major abdominal surgery | Continuous ICG infusion (Fick principle), pulmonary artery thermodilution | Major abdominal surgery ± dopexamine | 21 | Baseline; 6 h; 24 h | Total hepatovisceral blood flow; splanchnic oxygen delivery/extraction | None | Hepatovisceral flow increased 1.0→1.2 L/min/m² (+20%); fractional splanchnic flow remained 24–27% of cardiac output; oxygen extraction decreased 31→24%; oxygen consumption unchanged | Major abdominal surgery produces spontaneous moderate postoperative hepatovisceral hyperperfusion with preserved regional oxygen metabolism. Additional dopexamine does not further augment macrovascular hepatovisceral perfusion. | RoB 2 | Low |

Table S1. Relevant articles filtered through the selection protocol.
